# 

**Published:** 1990-06

**Authors:** 


### WEST OF ENGLAND MEDICAL JOURNAL

**JUNE 1990** **Volume 105(ii)**

Copyright © West of England Medical Journal Co Ltd

**Contents**

SCIENTIFIC PAPERS

39 **Cerebellar Glioblastoma Multiforme; A Report of Two Cases and Review of the Literature**
T. Z. Aziz and M. Stoddart

42 **Intra-ocular Lenses for Cataract Patients**
N. L. Dallas

43 **Pulmonary uptake of MDP**
E. R. Davies
P. Goddard, J. Bell, P. Jackson, E. Pitcher,
J. A. Bullimore, E. R. Davies

47 **Intra-operative Ultrasound in the Surgery of Insulinomas**
C. A. C. Clyne, W. J. Greene and R. B. Paisey

MEDICAL HISTORY

48 **Some Plymouth Worthies (Part 2)**
M. Reilly

51 **The Medicine of the People**
A. Chapman

54 **Launch of the 'New Journal'**

LEISURE

55 **A West Country Composer**
A. Dowling

57 **Dartmoor: A Place to Explore**
R. Feneley

CORRESPONDENCE

60 **Correspondent's Column**—Get-up-and-go Hospitals and Hotels, Aversion Therapy for the Football Hooligan, A Pathological Rose by Any Other Name.

61 **Letter to the Editor**—A New Approach to Medical Records

REPORTS OF MEETINGS

62 **Symposium—Some Recent Advances in Hepatobiliary Disease and in the Clinical Aspects of Bile Acids**, Held at the Bristol Royal Infirmary, September 1989

65 **South West Orthopaedic Club** Spring Meeting held at Cheltenham, 1989

67 **Surgical Club of South West England** Spring Meeting held at Taunton, 1989

*"What I know remains unknown, unless by me to others shown.* **Lucilius (180–102 BC)**

